# IGF-I increases markers of osteoblastic activity and reduces bone resorption via osteoprotegerin and RANK-ligand

**DOI:** 10.1186/1479-5876-11-271

**Published:** 2013-10-25

**Authors:** Lucia Guerra-Menéndez, Maria C Sádaba, Juan E Puche, Jose L Lavandera, Luis F de Castro, Arancha R de Gortázar, Inma Castilla-Cortázar

**Affiliations:** 1 Department of Medical Physiology, Universidad CEU San Pablo, Institute of Applied Molecular Medicine (IMMA), School of Medicine, Room D-201. C/ Boadilla del Monte s/n, km 5,3, 28668 Madrid, Spain; 2 Department of Histology, Universidad CEU San Pablo, Institute of Applied Molecular Medicine (IMMA), School of Medicine, Madrid, Spain

**Keywords:** IGF-I, GH, Bone metabolism, Gene expression, Osteocalcin, Osteoprotegerin, Parathormone, RANKL, IGFBP-4, IGFBP-5

## Abstract

**Background:**

Bone is one of the major target tissues for Insulin-like Growth Factor I (IGF-I). Low doses of IGF-I were able to improve liver-associated osteopenia. In the present work, a model of partial IGF-I deficiency was used in order to provide insight into the mechanisms of the beneficial actions of IGF-I replacement therapy in bone.

**Methods:**

Several proteins involved in osteoblastic/osteocyte and osteoclastic differentiation and activity were studied in the three experimental groups: control (CO) group (wild type mice, *Igf*^+/+^, n = 10), heterozygous *Igf*^+/-^ group with partial IGF-I deficiency (Hz, n = 10), and heterozygous *Igf*^+/-^ mice treated with IGF-I for 10 days (Hz + IGF-I, n = 10).

**Results:**

Data in this paper confirm that the simple partial IGF-I deficiency is responsible for osteopenia, determined by densitometry and histopathology. These findings are associated with a reduced gene expression of osteoprotegerin, sclerostin, calcitonin receptor (CTR), insulin-like growth factor binding protein 5 and RUNX2. IGF-I replacement therapy normalized CTR gene expression and reduced markers of osteoclastic activity.

**Conclusions:**

Low doses of IGF-I constituted a real replacement therapy that normalized IGF-I serum levels improving the expression of most of these proteins closely involved in bone-forming, and reducing bone resorption by mechanisms related to osteoprotegerin, RANKL and PTH receptor.

## Background

Bone is one of the major target organs for insulin-like growth factor I (IGF-I) [1-4], an anabolic hormone produced mainly by the liver upon growth hormone (GH) stimulation [5-7]. Liver cirrhosis is associated with osteopenia and low levels of IGF-I [8-10], constituting a well established condition of IGF-I deficiency [11,12]. In advanced liver cirrhosis, IGF-I serum levels decrease as a result of diminished hepatocellular biosynthetic function and progressive loss of GH receptors on hepatocytes [13,14].

However, the pathogenesis of osteopenia in liver cirrhosis is not fully understood, although malabsorption, malnutrition, vitamin D deficiency, reduced levels of sexual hormones and alcohol toxicity appear to be some of the factors involved in altered bone metabolism [15-19].

Several years ago, our team showed that low doses of IGF-I were able to correct osteopenia associated to experimental cirrhosis [20], suggesting a role for IGF-I deficiency in the genesis of osteopenia and the possible therapeutic effect of IGF-I in this condition. Nevertheless, the mechanisms of the beneficial actions of IGF-I replacement therapy on bone are not entirely known yet.

In order to gain more insight into the mechanisms underlying the osteopenia related to low levels of IGF-I, we appealed to an animal model of “IGF-I partial deficiency” recently characterized and proposed as a more suitable animal model to mimic recognizable syndromes associated to human conditions of IGF-I deficiency [21]. Three experimental groups were included in the present study: control (CO) group (wild type mice *Igf*^+/+^ treated with vehicle for 10 days), Hz (heterozygous group, *Igf*^+/-^ mice with partial IGF-I deficiency treated with vehicle for 10 days) and Hz + IGF-I group (heterozygous *Igf*^+/-^ mice treated with 2 μg/100 g body weight/day, for 10 days).

The aim of the present work was to study the effect of low doses of IGF-I on bone in this animal model, by determining the following parameters: 1) bone weight, morphometry, densitometry and cortical thickness in histological preparations; 2) gene expression of IGF-I, and GH and IGF-I receptors in bone; 3) gene and protein expression of key molecules involved in osteocyte or osteoblastic differentiation and activity, such as osteoprotegerin (OPG), sclerostin (SOST), insulin-like growth factor binding protein-5 (IGFBP-5), runt-related transcription factor 2 (RUNX2), calcitonin receptor (CTR) [22-25]; 4) gene and protein expressions related to osteoclastic activity or inhibition, such as insulin-like growth factor binding protein-4 (IGFBP-4), parathormone receptor-1 (PTHR1), receptor activator for nuclear factor κ B ligand (RANKL) [26-30]. In addition, both IGF-I and IGFBP-3 serum levels as well as serum parameters of osteoblastic or osteoclastic activities were also assessed in the three experimental groups.

## Methods

### Animals and experimental design

Experimental model was established and characterized as previously reported [21]. Briefly, IGF-I heterozygous mice were obtained by crossing transgenic mice, line 129SV and Igf1tm1Arge [31]. Animals were housed in cages placed in a room with a 12 hours light/12 hours dark cycle, and constant humidity (50-55%) and temperature (20–22°C). Food (Teklad Global 18% Protein Rodent Diet, Harlan Laboratories, Spain) and water were given *ad libitum*. All experimental procedures were performed in compliance with The Guiding Principles for Research Involving Animals and approved by the Bioethical Committee from the University CEU San Pablo (Madrid, Spain).

For genotyping of mice by PCR analysis (Applied Biosystems, 2720 Thermal Cycler, Spain), DNA was extracted from a piece of tail and specific primers were used to identify both *Igf1* and *Neo* genes (Extract-N-Amp TM Tissue PCR KIT, Sigma, USA).

Three groups of male mice 20 days old were included in the experimental protocol: control group of wild type animals *Igf*^+/+^ (CO), *Igf*^+/-^ animals with heterozygous IGF-I expression (Hz), and *Igf*^+/-^ animals with heterozygous IGF-I expression, which were subcutaneously treated with IGF-I (2 μg/100 g body weight/day, for 10 days). Both control and heterozygote groups received vehicle (succinate buffer, subcutaneously) in parallel, n = 10 each group.

On the 11^st^ day (the day after the last IGF-I injection), blood was obtained from submandibular vein and thereafter animals were sacrificed by cervical dislocation. Femurs and tibias were carefully dissected out and weighted (Denver Instrument, Germany). Samples from right tibia were processed for histological examination. Samples from left femur were immediately frozen by immersion in liquid N_2_ and stored at -80°C until analysis of gene expressions. Densitometry and bone morphometric studies were performed in the right femur. Serum was stored at -20°C.

### Analytical methods in serum

Alkaline phosphatase and glucose was determined in serum by routine laboratory methods using an autoanalyzer (Hitachi-Cobas Integra 400 plus, Roche Diagnostics, Spain). Serum levels of Osteocalcine, Osteoprotegerin and Leptin were assessed by Luminex (X-Map Technology), using specific commercial assay systems following protocol instructions (Millipore, USA). IGF-I (Mediagnost, Germany) and IGFBP-3 (Bionova, Spain) levels were determined in serum by ELISA.

### Morphological, immunohistochemical and densitometry parameters on bone

#### Cortical thickness and histopathological evaluation

Histopathological analyses were carried out in tibias, which were fixed in 4% paraformaldehyde diluted in PBS solution for 24 hours. Once they were properly fixed, they were included in ethanol 70%. For demineralization, it was used EDTA 5% in movement. Finally, the samples were embedded in paraffin using the automatic equipment (Leica TP 1020, Leica, Switzerland). Longitudinal sections (4 μm-thick, Reichert-Jung 2030 Biocut Microtome, Leica, Switzerland) were stained with hematoxylin-eosin. Morphometrical measurements (cortical thickness) were made by two observers at three points of diaphysis from each section using a light microscope (Leica, Switzerland). The arithmetical mean was used as final measure.

### Immunohistochemistry analyses and images processing

Immunohischemical studies were performed in order to spatially localize the related molecules within the cortical diaphysis. Samples were deparaffinized with HEMO-De (Scientific Safety Slovents, USA) and rehydrated in ethanol and PBS. Next, they were incubated with 0.3% hydrogen peroxide (Merck, Spain) in PBS for 30 min for inhibition of endogen peroxidase. Retrieval of antigen was induced with pepsin (Zymed, USA). Consecutive sections were incubated overnight at 4°C with different rabbit antibodies: rabbit Anti-IGF-I (1:50, Abcam, UK), rabbit Anti-IGF-IR (1:50, Abcam, UK), mouse Anti-GHR (1:50, Santa Cruz Biotechnology, USA), rabbit Anti-RANKL (1:500, Santa Cruz Biotechnology, USA), rabbit Anti-RANKL (1:500, Santa Cruz Biotechnology, USA), rabbit Anti-OPG (1:500, Santa Cruz Biotechnology, USA), rabbit Anti-CTR (1:100, Santa Cruz Biotechnology, USA), rabbit Anti-PTHR1 (1:50, Santa Cruz Biotechnology, USA), goat Anti-SOST (1:20, Santa Cruz Biotechnology, USA), to analyzed the osteoblatic and osteoclastic activity. After washing, slides were incubated for twenty minutes at room temperature with the biotin anti-rabbit/mouse complex (Histostain®-SP Broad Spectrum, Zymed, USA), except those labeled with SOST antibody that were developed with biotin anti goat IgG (Abcam, UK). Then, staining was performed incubating with streptavidin/horseradish peroxidase conjugated for ten minutes and diaminobenzidine (Sigma, USA), as chromogenic substrate for up to 5 minutes. Finally, samples stained with hematoxilin and covered with DEPEX medium.

Digital images of tibia sections were captured using Leica DFC345 FX® and a Leica DFC 425 camera (Leica, Switzerland). Three regions per sample were measured to evaluate immunohistochemical changes by using an image analysis Software (Leica MMAF 1.4 MetaMorph), which reported mean values of optical density (O.D.) for each sample.

### Morphological parameters and densitometry of bone

Femur length was measured from the major trochanter to the end of the distal epiphysis. All measurements were performed with a precision caliper, Vernier® (±0.005 mm).

Bone density from whole right femur was determined by Dual-Energy X-ray absorptiometry using PIXIMus I equipment (Lunar, USA) and thus expressed as the amount of mineralized tissue in the area scanned (g/cm^2^).

### Gene expression studies

#### Total RNA extraction, reverse transcription (RT-PCR) and quantitative real time (qPCR) PCRs

Left femur was cryopreserved in RNAlater (Qiagen-Izasa, Spain) after extracting bone marrow by NaCl 0.9% perfusion of medullary cavity with a syringe to exclude any interference of these cells on the PCR results. Then, they were homogenized with TRIzol reagent (Invitrogen, UK) by Tissue Lyser LT (Qiagen-Izasa, Spain) and RNA was extracted and further purified using the QIAGEN RNeasy Mini Kit including digestion with RNase-free DNase, following the manufacturer’s instructions. RNA quality was checked by the A260:A280 ratio and with the Bioanalyzer 2100 (Agilent Technologies Inc., USA). Purified RNA was then converted to cDNA by using the RNA-to-DNA EcoDryTM Premix (Clonetech Labs, USA) for qPCR assays.

Quantitative real time PCR assays were performed in a 3100 Avant Genetic Analyzer (Applied Biosystems Hispania, Spain).The thermal profile consisted on an initial 5 min melting step at 95°C followed by 40 cycles at 95°C for 10 s and 60°C for 60 s.

We used specific Taqman® probes for IGF-I (Mm00439560_m1), IGFBP-4 (Mm00494922_m1), IGFBP-5 (Mm00516037_m1), GHR, (Mm004390093_m1), IGF-IR(Mm00802831_m1), and gene expression related to osteoblastic and osteoclastic activity such as, CTR (Mm00432271_m1), SOST (Mm00470479_m1), PTHR1 (Mm00441046_m1), Runx2 (Mm00501584_m1), RANKL (Mm 00441908_m1), and OPG (Mm00435452_m1), supplied by Applied Byosistems.

The relative mRNA levels of the genes of interest were normalized to 18S expression using the simplified comparative threshold cycle delta, cycle threshold (CT) method [2^-(ΔCT gene of interest - ΔCT 18S)^].

### Statistical analysis

All data represent mean ± SEM. Statistical analysis was performed on SPSS 17 (Statistical Package for Social Sciences, USA). Significance was estimated by the U-Mann–Whitney test or, when appropriated, by analysis of variance (ANOVA). Correlation between IGF-I and weight was analyzed by Spearman test. Differences were considered significant at a level of p < 0.05.

## Results

### Normalization of IGF-I serum levels with low doses of this hormone

**Figure 1:**
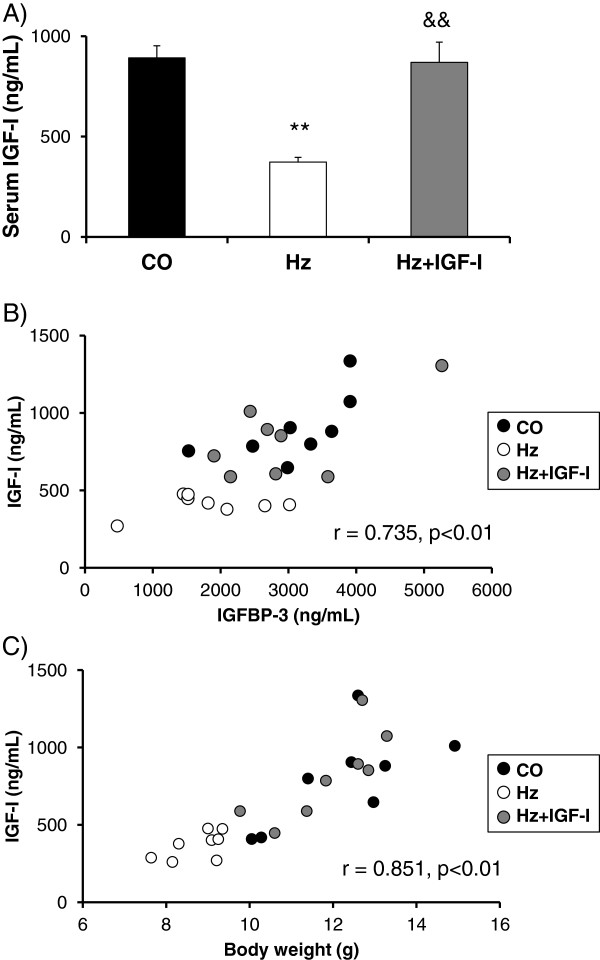
**Serum IGF-I levels and their correlations with IGFBP-3 and body weight. A**. Serum levels of IGF-I in controls (CO) mice with partial IGF-I deficiency (Hz) and Hz treated with low dose of IGF-I for 10 days. **B**. Direct and significant correlations between IGF-I and IGFBP-3. Data obtained from animals from the three experimental groups (n = 8 each group). **C**. Correlation between IGF-I and body weight. Data obtained from animals from the three experimental groups (n = 8 each group). **p < 0.01 CO vs Hz. &&p < 0.01 Hz vs Hz + IGF-I.

Accordingly to previous data [21], heterozygous (Hz) animals showed significantly lower levels of serum IGF-I serum as compared to control group (Hz: 372.65 ± 23.52 vs CO: 891.93 ± 60.51 ng/mL, p < 0.01). Interestingly, low doses of IGF-I were able to restore normal circulating levels of IGF-I (Hz + IGF-I: 869.42 ± 101.22 ng/mL, p < 0.01), p = n.s. vs CO, p < 0.01 vs Hz), acting as a real replacement therapy (Figure 1A).

In addition, IGF-I deficient animals (Hz) showed a significant lower IGFBP-3 serum levels (CO: 3,395.54 ± 298.78; Hz: 1,498.22 ± 201.54 ng/mL, p < 0.05) that IGF-I therapy normalized (Hz + IGF-I: 2,597.37 ± 389.05 ng/mL, p = n.s. vs CO). A direct and significant correlation between IGF-I and IGFBP-3 serum levels was found (Spearman Rho, r = 0.735, p < 0.01): Figure 1B.

Of interest, these low doses of IGF-1 did not induce hypoglycemia (CO: 151.14 ± 5.73 vs Hz + IGF-I: 143.82 ± 16.71, p = n.s.) or any other remarkable side effect.

### Effects of IGF-I replacement therapy on body weight, and bone morphometry and densitometry

In accordance with reported findings [21], a significant diminution of body weight was found in animals with partial IGF-I deficiency (Hz group) as compared to control group. Interestingly, exogenous administration of low doses of IGF-I were able to normalize body weight in IGF-I deficient mice (data not shown). A significant and direct correlation between body weight and IGF-I circulating levels was observed (r = 0.851, p < 0.01): Figure 1C.

Table 1 (upper side) summarizes femur weight and morphometric data. At the end of the study, femur weight was reduced in IGF-I deficient animals as compared with controls, but not in those treated with IGF-I (Hz + IGF-I, p = n.s. vs CO). Femur length, transversal diameter and surface (mm^2^) were reduced but did not reach statistical significance as compared to controls.

**Table 1:** Effect of IGF-I treatment on bone morphometry and serum biochemistry

	**CO**	**Hz**	**Hz + IGF-I**
	**(n = 10)**	**(n = 10)**	**(n = 10)**
Femur weight (mg)	33.10 ± 2.45	25.50 ± 2.40*	30.16 ± 2.84
Length (mm)	10.15 ± 0.44	9.25 ± 0.85	9.43 ± 0.63
Transversal diameter (mm)	1.05 ± 0.05	1.00 ± 0.05	1.02 ± 0.03
Surface (mm^2^)	10.60 ± 0.22	9.65 ± 1.14	9.64 ± 0.57
Alkaline phosphatase (U/dL)	378.14 ± 21.37	321.15 ± 19.92*	349.76 ± 13.07
Osteocalcin (ng/mL)	191.71 ± 5.41	164.81 ± 11.73*	185.14 ± 22.82
Osteoprotegerin (ng/mL)	3.15 ± 0.56	2.32 ± 0.28	3.31 ± 0.77
Leptin (ng/mL)	3.20 ± 0.35	2.61 ± 0.27	3.00 ± 0.45

*p < 0.05 vs CO.

**Figure 2:**
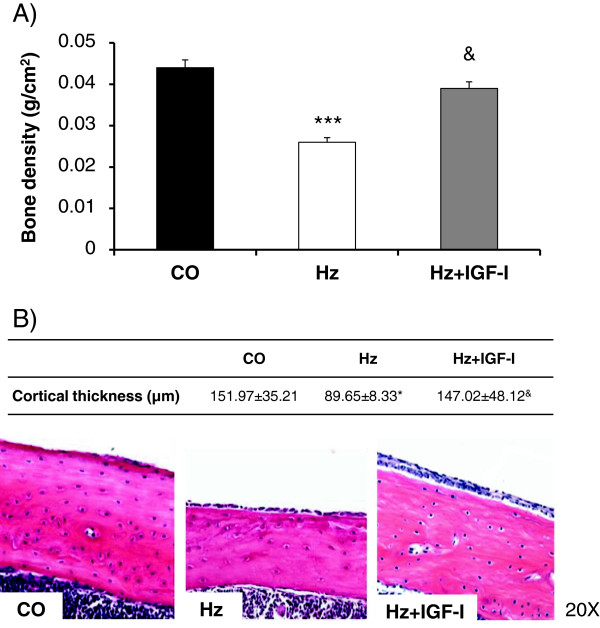
**Parameters of bone quality. A**. Bone mass by densitometry (g/cm^2^) in the three experimental groups. **B**. Bone cortical thickness was lower in Hz mice as compared to controls (CO) and Hz treated with low doses of IGF-I (Hz + IGF-I), determined by H&E. *p < 0.05 CO vs Hz, ***p < 0.001 CO vs Hz, &p < 0.05 Hz vs Hz + IGF-I.

However, IGF-I deficient group showed significantly lower bone mineral density as compared with controls (by 60%) (CO: 0.044 ± 0.005, Hz: 0.026 ± 0.001 g/cm^2^, p < 0.05), that IGF-I replacement therapy was able to prevent to a significant extent (up to 88%) (0.039 ± 0.006, p < 0.05 vs Hz) (see Figure 2A).

In addition, as it is showed in Figure 2B, cortical bone thickness was significantly lower in IGF-I deficient animals as compared with controls (p < 0.05), and IGF-I therapy induced a complete prevention of this decrease (p < 0.05 vs Hz, p = n.s. vs CO).

### Effects of IGF-I replacement therapy on serum parameters

Table 1 (lower side) also summarizes serum levels alkaline phosphatase, osteocalcin, osteoprotegerin and leptin. Although no significant differences were found in any of these serum parameters between the three experimental groups, in this series of young mice (31 days old), trends are consistent with reported results in the present study: increases in osteoblastic activity and reductions in osteoclastic activation.

### Gene expression of IGF-I, and GH and IGF-I receptors in bone

Accordingly to previous data [21], bone gene expression of IGF-I was significantly reduced in animals with partial IGF-I deficiency (CO: 1.00 ± 0.13; Hz: 0.30 ± 0.15 relative mRNA expression, p < 0.05 vs CO). The exogenous administration of IGF-I did not modulate IGF-I gene expression in these animals with systemic IGF-I gene disruption (Hz + IGF-I: 0.50 ± 0.17 relative mRNA expression, p < 0.05 vs CO, p = n.s. vs Hz).

Gene expression of IGF-I receptor (IGF-IR) in bone showed similar values in control and Hz groups, (CO: 1.00 ± 0.40; Hz: 0.92 ± 0.55 relative mRNA expression). However, IGF-I replacement therapy significantly reduced this value (Hz + IGF-I: 0.37 ± 0.15 relative mRNA expression, p < 0.05 vs CO and Hz groups). Consistently, the administration of IGF-I significantly reduced the protein expression of IGF-IR (CO: 0.42 ± 0.09; Hz: 0.41 ± 0.06; Hz + IGF-I: 0.31 ± 0.08 O.D., p < 0.05 vs CO and Hz groups).

On the other hand, both groups of IGF-I deficient animals expressed significant lower levels of GHR gene expression (Hz: 0.18 ± 0.09, Hz + IGF-I: 0.29 ± 0.12 relative mRNA expression, p = n.s. Hz vs Hz + IGF-I) compared to controls (CO: 1.00 ± 0.17 relative mRNA expression, p < 0.05 CO vs both heterozygous groups).

### Gene and protein expressions of molecules involved in osteoblastic, osteocyte or osteoclastic differentiation and activities

**Figure 3:**
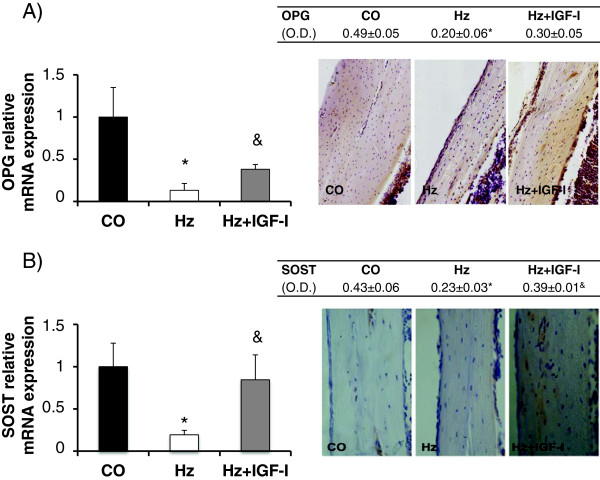
**Osteoprotegerin and sclerostin expressions. A**. Bone gene and protein expression of osteoprotegerin (OPG) and **B**. Bone gene and protein expression of sclerostin (SOST). *p < 0.05 vs CO, &p < 0.05 vs Hz. O.D., optical density.

Osteoprotegerin (OPG) is secreted by osteoblasts and inhibits bone resorption reducing both osteoclastic differentiation and activity [32,33]. Partial IGF-I deficiency was associated with a significant reduction of OPG gene expression (Figure 3A left panel) that the exogenous administration of IGF-I partially restored (CO: 1.00 ± 0.35, Hz: 0.13 ± 0.08, Hz + IGF-I: 0.38 ± 0.06 relative mRNA expression, p < 0.05 Hz vs CO, p < 0.05 Hz vs Hz + IGF-I, p = n.s. CO vs Hz + IGF-I). Immunohistochemistry analyses of OPG showed similar findings: Figure 3A (right panel).

In addition, sclerostin gene expression (SOST), the most reliable marker of osteocytes, was significantly diminished in Hz group as compared to control (CO: 1.00 ± 0.28, Hz: 0.19 ± 0.05 relative mRNA expression, p < 0.05) and IGF-I therapy normalized its expression (Hz + IGF-I: 0.84 ± 0.29 relative mRNA expression, p < 0.05 vs Hz, p = n.s. vs CO). Similar data were found in the immunohistochemistry study of sclerostin: Figure 3B (right panel).

**Figure 4:**
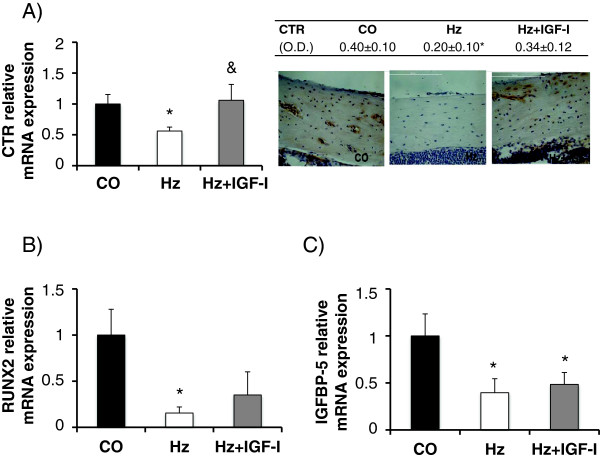
**Calcitonin receptor, RUNX2 and IGFBP-5 expressions. A**. Bone gene and protein expression of calcitonin receptor (CTR). **B**. Gene expression of RUNX2 (a marker of early osteoblastogenesis), and **C**. Gene expression of IGFBP-5 (a promoter of bone IGF-I activities). *p < 0.05 vs CO. O.D., optical density.

On the other hand, gene and protein expressions of calcitonin receptor, hormone that promotes osteoblastic activity and bone mineralization [34], were significantly lower in Hz group as compared to controls (see Figure 4A). Regarding gene expression (left 4A panel), we observed significantly lower levels in heterozygous group (CO: 1.00 ± 0.15, Hz: 0.56 ± 0.06 relative mRNA expression, p < 0.05 CO vs Hz), while no significant differences were found between control and heterozygous mice treated with IGF-I replacement therapy (Hz + IGF-I: 1.06 ± 0.57 relative mRNA expression). Consistently, immunohistochemistry analyses (right 4A panel) showed that calcitonin receptor was significantly decreased in untreated heterozygous group compared to control animals (p < 0.05 CO vs Hz) whereas no statistical differences were found in heterozygous treated mice.

RUNX2 (Runt-related transcription factor 2) is considered a good marker of early osteoblastogenesis and a promoter of bone differentiation and formation [23]. Partial IGF-I deficiency was associated to a significant reduction of RUNX2 gene expression (CO: 1.00 ± 0.28, Hz: 0.15 ± 0.07 relative mRNA expression, p < 0.05) that IGF-I therapy was able to correct partially (Hz + IGF-I: 0.35 ± 0.15 relative mRNA expression, p = n.s. vs control group): Figure 4B.

In addition, as compared to controls, both IGF-I deficient groups showed a lower expression of IGFBP-5, a binding protein that promotes bone IGF-I activities [27,32,33,35], that the exogenous administration of IGF-I did not modulate (CO: 1.00 ± 0.23, Hz: 0,39 ± 0.15, Hz + IGF-I: 0.48 ± 0.13 relative mRNA expression, p < 0.05 both groups vs CO): Figure 4C.

**Figure 5:**
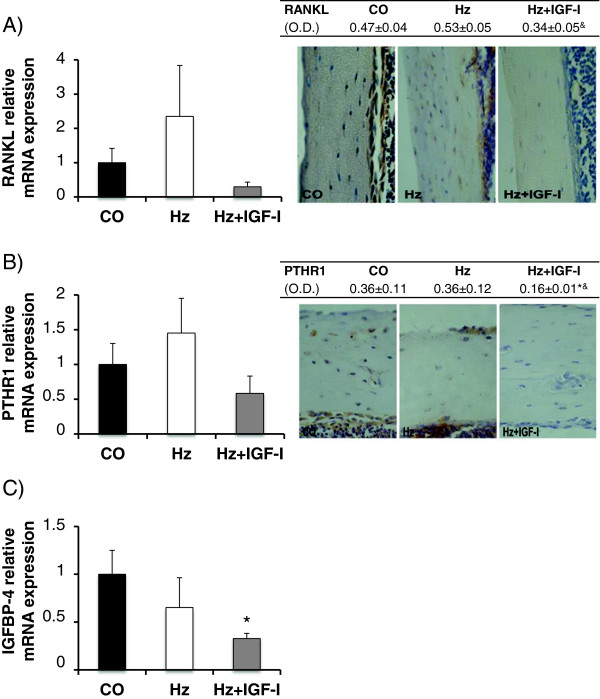
**RANKL, PTHR1 and IGFBP-4 expressions. A**. Bone gene and protein expression of RANKL. **B**. Bone gene and protein expression of PTHR1. **C**. Bone gene expression of IGFBP-4. *p < 0.05 vs CO, &p < 0.05 vs Hz. O.D., optical density.

Gene expression of RANKL in untreated heterozygous group was increased as compared to control group (CO: 1.00 ± 0.42, Hz: 2.35 ± 1.40 relative mRNA expression). IGF-I exogenous administration was able to reduce its expression (Hz + IGF-I: 0.29 ± 0.14 relative mRNA expression (Figure 5A, left panel). Accordingly, protein expression was significantly higher in partial IGF-1 deficient animals (p < 0.05) and the replacement therapy was able to normalize it (p < 0.05 vs Hz group): Figure 5A.

Partial IGF-I deficiency was also associated to an increase of PTH receptor (PTHR1), although without reaching statistical significance (CO: 1.00 ± 0.30, Hz: 1.45 ± 0.50 relative mRNA expression). Interestingly, IGF-I replacement therapy was able to reduce its gene (Hz + IGF-I: 0.55 ± 0.25 relative mRNA expression) and protein expressions (see Figure 5B).

Finally, IGFBP-4 is an IGF-I binding protein that inhibits IGF-I actions in bone [26]. In the present study, IGF-I deficient mice showed a tendency to decrease IGFBP-4 gene expression in bone (CO: 1.00 ± 0.25, Hz: 0.65 ± 0.31 relative mRNA expression), that IGF-I therapy made even greater reaching statistical significance (Hz + IGF-I: 0.33 ± 0.05 relative mRNA expression, p < 0.05 vs CO): Figure 5C.

## Discussion

In the last years, IGF-I treatment is being either tested in or proposed for a wide range of pathological circumstances, as Laron syndrome, chronic liver disease, intrauterine growth restriction, insulin resistance and diabetes, neurological disorders (Alzheimer’s disease and amyotrophic lateral sclerosis) and stroke, cystic fibrosis, wound healing, burns, etc. [36-48]. However, in our opinion, only those states correlating with low levels of IGF-I may benefit from a real replacement therapy, thus avoiding potential adverse effects [49].

Data in this work provide evidences that the simple partial IGF-I deficiency is associated with decreased bone weight, cortical thickness and densitometry and that these bone deficits can be corrected by a short-term course of IGF-I at low doses, without hypoglycemia or any other adverse effects.

This study fits in a series of works to elucidate the mechanisms of the beneficial actions of IGF-I therapy in liver cirrhosis [13,14,39,50-57], a condition of IGF-I deficiency [11,12]. In cirrhotic patients, prevalence of osteopenia is significantly higher than in age-matched normal population [15]. Consequently, these patients are exposed to an increased risk of bone fractures, which are a source of morbidity in advanced stages of the disease and after liver transplantation [15,58]. The pathogenesis of osteopenia in chronic liver disease is not fully understood, since many factors have been involved (malabsorption, malnutrition, vitamin D deficiency, reduced level of sexual hormones or alcoholic toxicity) [15-20].

In order to distinguish the involvement of all these factors, we resorted to an animal model of partial, and systemic, IGF-I deficiency [21], where the mechanism responsible for osteopenia has to be exclusively the partial IGF-I deficiency. In this sense, for this protocol, mice of 20 days old were chosen in order to study from early ages the consequence of the single IGF-1 deficiency on bone, since, as previously reported, the significant reduction of IGF-1 circulating levels are persistent along the life in this experimental model [21].

First of all, in the animal model used in this work, IGF-I serum levels and bone IGF-I gene expression were found reduced in heterozygous mice associated to diminished bone weights, densitometry values and cortical thickness (see Table 1 and Figure 2B). These results demonstrated that the only partial IGF-I deficiency is responsible for osteopenia. These changes in bone are similar to those previously described in rats with liver cirrhosis induced by CCl_4_ exposure [20]. This conclusion about the role of IGF-I in this bone disturbance is reinforced since IGF-I replacement therapy (at the same doses used in cirrhotic animals) is able to reverse it.

Previously, we described that the malnutrition occurring in cirrhotic rats had a greater impact on striated muscle and fat than on bone [20]. Results in this work confirm that the IGF-I deficiency seems to be a relevant causal factor of osteopenia in cirrhosis by the decreased biosynthetic capability of the liver.

On the one hand, results in this and previous studies [20] suggest that osteopenia is the consequence of an increase of bone resorption. In untreated cirrhotic rats a significant increase in urinary excretion of deoxypyridinoline cross-links indicated a enhanced bone resorption and osteoclastic activity, that IGF-I replacement therapy was able to normalize [20]. These findings were in agreement with *in vitro* studies using bone tissue cultures in which IGF-I was reported to both inhibit osteoclasts and interfere with osteoblast-derived factors that stimulate existing osteoclasts [59]. However, in those days, the mechanisms responsible were not fully ascertained.

**Figure 6:**
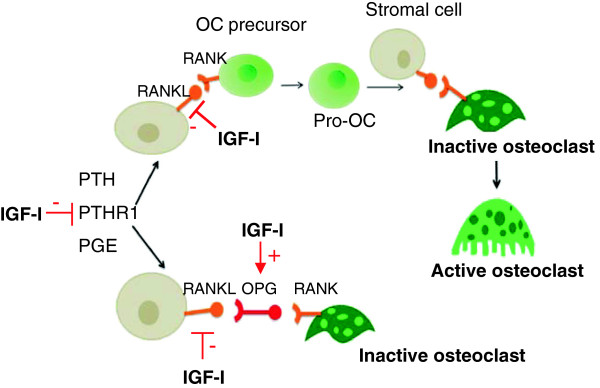
**Mechanisms of IGF-I actions on osteoclastic activation mediated by OPG and RANKL.** RANKL promotes osteoclastic differentiation and activity while OPG is able to avoid the link between RANK and RANKL.

One of the most relevant findings in this manuscript is the significant hypoexpression of osteoprotegerin (OPG) in IGF-I deficient mice, and its improvement by the exogenous administration of IGF-I (Figure 3A). OPG is a protein produced by osteoblasts that inhibits osteoclastic differentiation [60-62], thus preventing their differentiation by standing at the binding site of RANKL with its receptor [60,62]. Moreover, IGF-I deficient mice overexpressed RANKL while IGF-I replacement therapy normalizes its expression (Figure 5A). In addition, PTH and its receptor are involved in osteoclastic differentiation and activity [35,63]. In the present study, it was found that IGF-I exogenous administration reduced PTHR1 expression (Figure 5B). All these mechanisms, that may explain a reduction on bone resorption, are summarized in Figure 6.

On the other hand, the partial IGF-I deficiency was linked to a decrease of the following gene expressions: CTR, SOST, and IGFBP-5, all of them closely related, through distinct mechanisms, to the promotion of the osteoblastic activity or osteoclastic inhibition [39,60]. This outcome is consistent with previous data [64,65], where osteocyte PTH receptor 1 activation was shown sufficient to decrease SOST expression. Additionally, partial IGF-I deficiency was associated with bone hypoexpression of the RUNX2 gene, a marker of early osteoblastogenesis [58,61].

Interestingly, exogenous administration of IGF-I improved calcitonin receptor, SOST and RUNX2 expressions. IGFBP-5 has been described as an IGF-I carrier protein that promotes its actions in bone [27,32,33,35] and, although IGF-I replacement therapy did not module its hypoexpression, it could also be involved in the reduction of bone mass observed in these heterozygous mice. However, IGFBP-4 competes with IGF-I in its receptor and inhibits *in vitro* osteoblastic activity [26,35]. In the present study, IGF-I replacement therapy induced a significant decrease of IGFBP-4 gene expression, suggesting that this inhibitory mechanism may be contributing to the increase in bone mass described in this paper.

These findings are consistent with the known effects of IGF-I on bone both *in vivo *[3,66-68] and *in vitro*, where IGF-I enhances bone collagen and matrix synthesis and stimulates the replication of cells from the osteoblast lineage [66,68].

In summary, regarding the mechanisms involved in bone-formation, IGF-I therapy was able to significantly attenuate the decrease in bone weight and densitometry observed in Hz mice, restoring both densitometry values and histopathological parameters (cortical thickness), suggesting an increased bone-forming activity in the tibia in Hz + IGF-I mice as compared with Hz group. Accordingly, the exogenous administration of IGF-I, at low doses, normalized the calcitonin receptor, OPG and SOST gene expressions, while it reduced IGFBP-4 gene expression, a carrier protein that, as stated before, inhibits IGF-I activities in bone [27,32,33,35].

## Conclusion

In conclusion, partial IGF-I deficiency alone is responsible for osteopenia, characterized by reduced bone mass determined by densitometry and histology, associated with a reduced expression of several proteins involved in osteoblastic/osteocyte activity (OPG, SOST, CTR, IGFBP-5 and RUNX2) and with an overexpression of proteins promoting osteoclastic actions, providing at least two mechanisms possibly contributing to the observed reduction on bone mass in Hz mice. Low doses of IGF-I constitute an effective replacement therapy that normalizes IGF-I serum levels and modulate the expression of most of these proteins, increasing bone-formation and reducing bone resorption.

## Abbreviations

CO: Control; CT: Cycle threshold; CTR: Calcitonin receptor; GH: Growth hormone; GHR: Growth hormone receptor; Hz: Heterozygous; IGF-I: Insulin-like growth factor; IGF-IR: IGF-I receptor; IGFBP: Insulin-like growth factor binding protein; KO: Knockout; N.s: Not significant; OC: Osteocalcin; O.D: Optical density; OPG: Osteoprotegerin; PTH: Parathormone; PTHR1: PTH receptor 1; RANKL: Receptor activator for nuclear factor κ B ligand; RUNX2: Runt-related transcription factor 2; SEM: Standard error of mean; SOST: Sclerostin.

## Competing interests

The authors declare that they have no competing interests.

## Authors’ contributions

LG: Experimental procedures of the *in vivo* protocol, histological densitometry, PCR technique and data acquisition and analyses. MCS: Analytical procedures and data and statistic analyses. JEP: Data and statistic analyses, manuscript and figure editing, and critical review. JLL: Analytical procedures and data and statistic analyses. LFC: Densitometry and PCR assays. ARG: Histopathological studies. ICC: Hypothesis and protocol designs, data analyses, manuscript elaboration and critical review. All authors read and approved the final manuscript.
